# Structure and Interactions of the Human Programmed Cell Death 1 Receptor* [Fn FN2]

**DOI:** 10.1074/jbc.M112.448126

**Published:** 2013-02-15

**Authors:** Xiaoxiao Cheng, Vaclav Veverka, Anand Radhakrishnan, Lorna C. Waters, Frederick W. Muskett, Sara H. Morgan, Jiandong Huo, Chao Yu, Edward J. Evans, Alasdair J. Leslie, Meryn Griffiths, Colin Stubberfield, Robert Griffin, Alistair J. Henry, Andreas Jansson, John E. Ladbury, Shinji Ikemizu, Mark D. Carr, Simon J. Davis

**Affiliations:** From the ‡ Radcliffe Department of Medicine and; § Medical Research Council Human Immunology Unit, University of Oxford, John Radcliffe Hospital, Headington, Oxford OX3 9DU, United Kingdom,; the ¶ Department of Biochemistry, University of Leicester, Leicester LE1 9HN, United Kingdom,; the ‖ Institute of Organic Chemistry and Biochemistry, Flemingovo Namesti 2, 166 10 Prague 6, Czech Republic,; the ** Department of Biochemistry and Molecular Biology, University of Texas M. D. Anderson Cancer Center, Houston, Texas 77030,; ‡‡ UCB Pharma, Slough SL1 4EN, United Kingdom,; the §§ Systems Biology Research Centre, School of Life Sciences, University of Skövde, Box 408, Skövde, Sweden, and; the ¶¶ Division of Structural Biology, Graduate School of Pharmaceutical Sciences, Kumamoto University, 5-1 Oe-honmachi, Kumamoto 862 0973, Japan

**Keywords:** Cell Surface Protein, Nuclear Magnetic Resonance, Receptors, Signaling, Surface Plasmon Resonance (SPR), Affinity, Complex Formation, Thermodynamics

## Abstract

PD-1, a receptor expressed by T cells, B cells, and monocytes, is a potent regulator of immune responses and a promising therapeutic target. The structure and interactions of human PD-1 are, however, incompletely characterized. We present the solution nuclear magnetic resonance (NMR)-based structure of the human PD-1 extracellular region and detailed analyses of its interactions with its ligands, PD-L1 and PD-L2. PD-1 has typical immunoglobulin superfamily topology but differs at the edge of the GFCC′ sheet, which is flexible and completely lacks a C″ strand. Changes in PD-1 backbone NMR signals induced by ligand binding suggest that, whereas binding is centered on the GFCC′ sheet, PD-1 is engaged by its two ligands differently and in ways incompletely explained by crystal structures of mouse PD-1·ligand complexes. The affinities of these interactions and that of PD-L1 with the costimulatory protein B7-1, measured using surface plasmon resonance, are significantly weaker than expected. The 3–4-fold greater affinity of PD-L2 *versus* PD-L1 for human PD-1 is principally due to the 3-fold smaller dissociation rate for PD-L2 binding. Isothermal titration calorimetry revealed that the PD-1/PD-L1 interaction is entropically driven, whereas PD-1/PD-L2 binding has a large enthalpic component. Mathematical simulations based on the biophysical data and quantitative expression data suggest an unexpectedly limited contribution of PD-L2 to PD-1 ligation during interactions of activated T cells with antigen-presenting cells. These findings provide a rigorous structural and biophysical framework for interpreting the important functions of PD-1 and reveal that potent inhibitory signaling can be initiated by weakly interacting receptors.

## Teaser

**Background:** The inhibitory leukocyte receptor PD-1 binds two ligands, PD-L1 and PD-L2.

**Results:** Nuclear magnetic resonance analysis and rigorous binding and thermodynamic measurements reveal the structure of, and the mode of ligand recognition by, PD-1.

**Conclusion:** PD-L1 and PD-L2 bind differently to PD-1 and much more weakly than expected.

## Introduction

In recent years, PD-1 (programmed cell death 1) has emerged as one of the most important inhibitory molecules in the immune system. Its potent inhibitory activity became evident when mice ablated at the *Pdcd1* locus developed strain-specific autoimmunity: sporadic glomerulonephritis on a C57BL/6 background (1) and cardiomyopathy in BALB/c mice (2). Genetic studies in humans also emphasize its importance insofar as *PDCD1* gene polymorphisms were found to confer susceptibility to systemic lupus erythematosus, atopy, and rheumatoid arthritis (3–5). PD-1 is also responsible for the “exhausted” phenotype of antigen-specific T cells in animal models of chronic infection (6, 7) and in human immunodeficiency (8) and hepatitis (9, 10) virus infections (although the latter is disputed (11)). It has also been implicated in the *de novo* generation of regulatory T cells (12). Such effects have made PD-1 one of the most actively studied therapeutic targets in cancer immunotherapy; presently, four anti-PD-1 antagonists are in clinical trials (reviewed in Ref. 13). It is suggested that PD-1 inhibits signaling, in T cells at least, by recruiting the phosphatase SHP-2 to TCR^4^ microclusters during the early stages of immunological synapse formation, where it blocks on-going TCR signaling (14).

PD-1 expression is induced upon the activation of CD4^+^ T cells, CD8^+^ T cells, NKT cells, B cells, and monocytes (15), whereupon it binds two distinct ligands, PD-L1 (B7-H1 or CD274 (16, 17)) and PD-L2 (B7-DC (18, 19). PD-L1 is both constitutively and inducibly expressed by T and B cells, dendritic cells (DCs), macrophages, mesenchymal stem cells, and bone marrow-derived mast cells and on nonhematopoietic cells; PD-L2 expression is up-regulated on DCs, macrophages, and mast cells (reviewed in Ref. 15). PD-1 is a monomeric type I surface glycoprotein consisting of a single V-set immunoglobulin superfamily (IgSF) domain attached to a transmembrane domain and a cytoplasmic domain with two tyrosine-based signaling motifs. PD-1 is often assigned to the CD28 receptor family, mostly on the basis of functional similarities (*e.g.* see Ref. 20). However, PD-1 actually shares more structural homology with antigen receptors and CD8 and can be considered to be intermediate between the antigen receptors and CD28 family proteins, suggesting that a PD-1-like protein was a precursor of IgSF family signaling receptors (21). Like the ligands of CD28 and CTLA-4, PD-L1 and PD-L2 are B7 family proteins comprised of tandem V-set and C1-set IgSF domains. In addition to PD-1, PD-L1 binds B7-1, one of the ligands of CD28 and CTLA-4 (22, 23), potentially interlocking the PD-1 and CD28/CTLA-4 signaling pathways. Structures of mouse PD-1 complexed with human PD-L1 (24) and mouse PD-L2 (25) revealed that these proteins interact largely orthogonally via their GFCC′C″ β-sheets. The complex of mouse PD-1 and human PD-L1 (24) is highly reminiscent of V-set domain dimers in antigen receptors, suggesting how in *trans* interacting receptors could have evolved into in *cis* interacting IgSF dimers, or *vice versa* (21, 26).

Despite its considerable immunotherapeutic potential, we know relatively little about the structure and interactions of human PD-1. There are no published structures of ligand-bound or unbound forms of the receptor, and whereas relatively high avidities have been measured for the interactions of bivalent forms of PD-1 with its ligands (reviewed in Ref. 15), there have been no systematic measurements of the true affinities. Here, we present the structure of a soluble form of human PD-1 and map its interactions with PD-L1 and PD-L2 using nuclear magnetic resonance (NMR)-based approaches. The new structure helps to account for the distinct affinity and thermodynamic properties of PD-1 binding to PD-L1 and PD-L2. Measurements of the human and mouse affinities suggest that potent inhibitory signaling can be mediated by surprisingly weak interactions. Finally, we use simulations of signaling complex formation to explore the reasons why PD-1 might have two distinct ligands and to gauge the impact of PD-L1 binding to B7-1.

## EXPERIMENTAL PROCEDURES

### 

#### 

##### Expression and Purification of PD-1 and Its Ligands

The extracellular region of the mature form of human PD-1 (hPD-1; residues 14–130), with a Met added to the N terminus and a Cys to Ser mutation introduced at position 73 to aid expression and folding, was expressed in the form of untagged protein in inclusion bodies in *Escherichia coli* BL21 (DE3) pLysS cells using a modified pET vector (Novagen). Uniformly ^15^N-, ^15^N/^13^C-, and ^2^H/^13^C/^15^N-labeled hPD-1 was produced from cells grown in minimal medium containing [^15^N]ammonium sulfate and d-[^13^C]glucose, if required, as the sole nitrogen and carbon sources, and 100% D_2_O when appropriate. Refolding conditions were determined using the iFOLD System 2 Screen (Merck). The hPD-1-expressing cells were resuspended in 50 mm Tris-HCl, 50 mm NaCl, 1 mm Tris(hydroxypropyl)phosphine, 0.5 mm EDTA, 5% glycerol, pH 8.0, before passing twice through a cell disrupter at 30,000 p.s.i. The lysate was made up to 125 mm with non-detergent sulfobetaine 201 (Sigma) and mixed. The hPD-1-containing inclusion bodies were washed once with the above buffer containing 125 mm non-detergent sulfobetaine 201 and then three times with the same buffer without non-detergent sulfobetaine 201. Inclusion bodies were solubilized in 50 mm Tris-HCl, 200 mm NaCl, 2 mm EDTA, 6 m guanidine HCl, pH 8.0. hPD-1 was subsequently efficiently refolded by rapid dilution into 50 mm HEPES, pH 7.5, 500 mm l-arginine, 9 mm glutathione, 1 mm glutathione disulfide, 24 mm NaCl, 1 mm KCl. The refolding mixture was then concentrated by tangential flow filtration, and the refolded protein was purified using a 16/60 Superdex 200 gel filtration column (GE Healthcare). The authenticity of the refolded hPD-1 material was assessed by the comparison of its ^1^H NMR spectrum with the data obtained for the protein produced in a eukaryotic expression system. Unlabeled, soluble His_6_-tagged, and biotinylatable forms of human and mouse PD-L1 (hPD-L1 and mPD-L1) and PD-L2 (hPD-L2 and mPD-L2) were produced via stable expression in Chinese hamster ovary (CHO) cells, using approaches used previously (27–29).

##### NMR Spectroscopy

NMR spectra were acquired from 0.35-ml samples of 0.5 mm free hPD-1 and 0.2 mm hPD-1·hPD-L1 or hPD-1·hPD-L2 complex in a 25 mm sodium phosphate, 100 mm sodium chloride buffer at pH 6.4, containing 5% D_2_O, 95% H_2_O. All of the NMR data were collected at 25 °C on either 600- or 800-MHz Bruker Avance spectrometers equipped with triple resonance (^15^N/^13^C/^1^H) cryoprobes. A series of double and triple resonance spectra were recorded to determine essentially complete sequence-specific resonance assignments for hPD-1, as described previously (30–32). ^1^H-^1^H distance constraints required to calculate the structure of hPD-1 were derived from NOEs identified in NOESY, ^15^N/^1^H NOESY-HSQC, and ^13^C/^1^H HSQC-NOESY spectra, which were acquired with an NOE mixing time of 100 ms. The specific binding of either hPD-L1 or hPD-L2 to hPD-1 was monitored by changes induced in the positions of signals of ^2^H/^13^C/^15^N-labeled hPD-1 in three-dimensional TROSY-HNCO spectra (33). Residues involved in forming stable backbone hydrogen bonds were identified by monitoring the rate of backbone amide exchange in two-dimensional ^15^N/^1^H HSQC spectra of hPD-1 dissolved in D_2_O.

##### Structural Calculations

The family of converged hPD-1 structures was initially calculated using Cyana 2.1 (34), as described previously (35). The combined automated NOE assignment and structure determination protocol was used to automatically assign the NOE cross-peaks identified in two-dimensional NOESY and three-dimensional ^15^N- and ^13^C-edited NOESY spectra and to produce preliminary structures. In addition, backbone torsion angle constraints, generated from assigned chemical shifts using the program TALOS+ (36), and hydrogen bond constraints involving residues with slowly exchanging amide protons were included in the calculations. Subsequently, five cycles of simulated annealing combined with redundant dihedral angle constraints (Redac) (37) were used to produce the 52 converged hPD-1 structures with no significant restraint violations (distance violations <0.2 Å and dihedral angle violations <5°), which were further refined with two cycles of restrained molecular dynamics simulated annealing using AMBER (38). Initial energy minimization (2000 steps) was followed by 20 ps of simulated annealing in vacuum and three cycles of 20-ps simulated annealing using a generalized Born solvent model (39) with force constants of 30 kcal mol^−1^ Å^2^ for distance constraints (NOEs, hydrogen bonds, disulfide bridge), 1000 kcal mol^−1^ rad^−2^ for dihedral angle constraints, and 10 kcal mol^−1^ rad^−2^ for chirality constraints. The 35 structures with the lowest AMBER energy and with no distance constraint violation greater than 0.18 Å and dihedral angle constraint violation greater than 5° were selected. Analysis of the family of structures obtained was carried out using the programs Molmol, Molprobity, and PyMOL (40–42).

##### Analysis of NMR Binding Data

The minimal shift approach (43–45) was used to assess the changes in the positions of hPD-1 backbone signals (H^N^, N, and C′) resulting from the binding of hPD-L1 or hPD-L2. A detailed description of the exact procedure is published (35). To facilitate the identification of ligand binding sites on the surface of hPD-1, histograms of the actual and minimal combined shift *versus* the protein sequence were used to identify regions of the protein containing a number of significantly perturbed backbone signals. The affected residues within these regions were then assessed as possible interaction sites in the ligand-binding site by examination of the solution structure determined for hPD-1.

##### Surface Plasmon Resonance (SPR) Experiments

Binding experiments were carried out using surface plasmon resonance as implemented in the Biacore^TM^ 3000 (GE Healthcare). Affinity and kinetic analyses were performed at 37 °C in HBS-EP buffer (25 mm HEPES, pH 7.4, 150 mm NaCl, 3.4 mm EDTA, and 0.005% surfactant P20; GE Healthcare). For experiments to determine the binding affinity of human and mouse PD-1 for their ligands, biotinylated soluble forms of human and mouse PD-L1 and PD-L2 or control biotinylated protein (CD4) were indirectly immobilized to the sensor surface of SA sensor chips (GE Healthcare) via streptavidin to levels of ∼2000 response units (RU) as described previously (46). Soluble, monomeric forms of PD-1 were then injected over the immobilized ligands. Alternatively, human or mouse PD-1Fc fusion protein or control fusion protein (CD28Fc (47)) at 25 μg/ml in 10 mm sodium acetate, pH 4.5, was directly immobilized to the dextran matrix of research grade CM5 sensor chips (GE Healthcare) by amine coupling using the manufacturer's kit (GE Healthcare) and an activation time of 5 min, resulting in immobilization levels of ∼4000 RU. In this case, His_6_-tagged forms of hPD-L1 and hPD-L2 were injected over the immobilized PD-1Fc. For kinetic analyses in each orientation, the immobilization levels were lower, at ∼500–1200 RU. Equilibrium binding analysis was undertaken as described (28, 29). Briefly, serial dilutions of hPD-L1 or hPDL-2 or of PD-1 monomer (released by thrombin treatment of PD-1Fc fusion protein (47)) were injected simultaneously over flow cells containing directly immobilized PD-1Fc (or CD28Fc) or indirectly immobilized biotinylated hPD-L1 and hPD-L2 or control protein (CD4) at 25 and 37 °C. Injections were of 1-min duration, at a buffer flow rate of 10 μl/min, which was sufficient for binding to reach equilibrium. For the kinetic analyses, dissociation rates were measured as described (28, 29). The binding data were examined using BIAevaluation software (GE Healthcare), and affinity and kinetic parameters were derived using the curve fitting tools of Origin version 5.0 (MicroCal Software Inc., Northampton, MA).

##### Isothermal Titration Calorimetry (ITC)

ITC experiments were performed using the MCS or VP-ITC systems (MicroCal Software Inc.) as described (48, 49). In a typical experiment, hPD-L1 or hPD-L2 at 0.2 mm was added in 20 15-μl injections to a 0.02 mm solution of human PD-1Fc in the 1.463-ml calorimeter cell at the temperatures indicated. The resulting data were fitted as described (48) after subtracting the heats of dilution resulting from the addition of hPD-L1 or hPD-L2 to buffer and buffer to hPD-1Fc, determined in separate control experiments. Titration data were fitted using a non-linear least squares curve-fitting algorithm with three floating variables: stoichiometry, association constant (*K_a_*), and change of enthalpy on binding (Δ*H*_obs_). All binding data were analyzed by fitting the binding isotherm to a single independent binding site model using Origin software provided with the ITC. ITC allows for the complete thermodynamic characterization of an interaction based on the relationship, Δ*G* = −*RT* × ln(*K_a_*) = Δ*H*_obs_ − *T*Δ*S*, where *R* is the gas constant, *T* is the absolute temperature, and Δ*G*, Δ*H*_obs_, and Δ*S* are the standard free energy, observed enthalpy, and entropy changes going from unbound to bound states, respectively. All experiments were done in triplicate.

##### Simulations of PD-1·PD-L1 and PD-1·PD-L2 Complex Formation

The theoretical framework used to simulate the interactions of human PD-1 with its ligands and of human PD-L1 with B7-1 is analogous to that used previously to analyze costimulatory interactions at the synaptic interface between a naive/activated T cell and an immature/mature DC (50, 51). A description of the model is given in the supplemental Experimental Procedures. Parameter values for the molecular interactions and the expression levels used in the simulations are taken from the present study, whereas the values for diffusion and mobility used are those for costimulatory molecules (50).

## RESULTS

### Human PD-1 Extracellular Region Structure Determination

**FIGURE 1.**
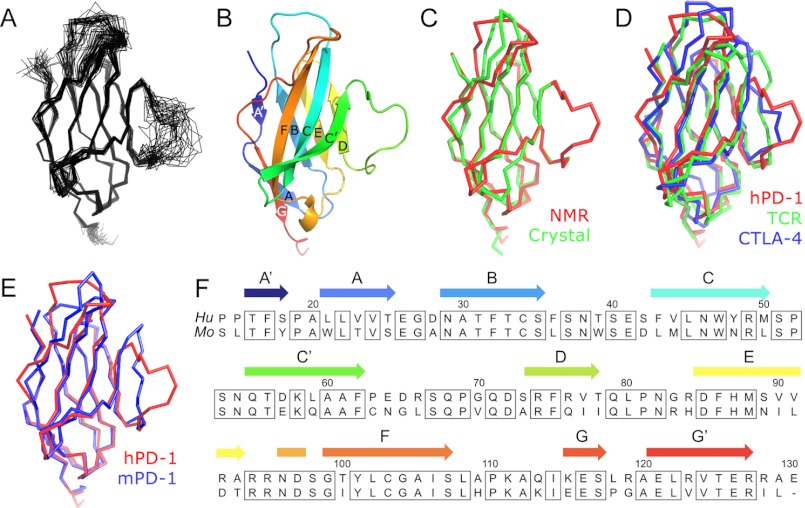
**Solution structure of the extracellular domain of human PD-1.** *A*, best fit superposition of the protein backbone for the 35 converged structures obtained for hPD-1. *B*, *ribbon representation* of the backbone topology of the structure closest to the mean, in the same orientation. *C*, comparison of the NMR-based (*red*) and crystal (*green*; PDB accession number 3RRQ) structures of hPD-1. *D*, comparison of the structures of hPD-1, TCRδ V-domain (PDB accession number 3OMZ), and CTLA-4 (PDB accession number 3OSK). *E*, comparison of the backbone topologies of human (*red*) and mouse (PDB accession number 1NPU; *blue*) PD-1. *F*, structure-based alignment of the sequences of human (*Hu*) and mouse (*Mo*) PD-1 (mature polypeptide numbering).

The authenticity of refolded, ^13^C/^15^N-labeled hPD-1 produced in bacteria (see “Experimental Procedures”) was confirmed by showing that its one-dimensional ^1^H NMR spectra was essentially indistinguishable from that of deglycosylated hPD-1 expressed in Chinese hamster ovary cells (data not shown). The thermal stability of this material, tested by differential scanning fluorimetry (data not shown), was sufficient for the acquisition of multiple three-dimensional NMR experiments at 25 °C. Comprehensive sequence-specific backbone and side chain resonance assignments were obtained using established triple resonance experiments (30–32). Resonance assignments obtained for protons were >96.4% complete, with only the two N-terminal residues (Met and Pro) and parts of several aromatic side chains remaining unassigned. The completeness of the ^15^N, ^13^C, and ^1^H resonance assignments allowed automated assignment of the NOEs identified in three-dimensional ^15^N/^1^H NOESY-HSQC, in ^13^C/^1^H HSQC-NOESY, and in the aromatic to aliphatic region of two-dimensional NOESY spectra using the CANDID protocol implemented in Cyana (34). This yielded unique assignments for 94.9% (2702 of 2847) of the NOE peaks observed, providing 1561 non-redundant ^1^H-^1^H distance constraints. Fifty-two satisfactorily converged hPD-1 structures were obtained from 100 random starting conformations using 1879 NMR-derived structural constraints (∼16 constraints/residue), which were further refined in AMBER by simulated annealing using a generalized Born solvent model (39). A final round of refinement yielded 35 structures with no distance violations of >0.18 Å (Fig. 1*a* and Table 1). The hPD-1 structures, NMR constraints, and resonance assignments have been deposited in the Protein Data Bank (PDB; accession number 2M2D) and BMRB database (accession number 18908).

**TABLE 1:** **NMR constraints and structural statistics for PD-1**

**No. of constraints used in structural calculations**	
Intraresidue NOEs	399
Sequential NOEs (*i*, *i* + 1)	432
Medium range NOEs (*i*, *i* > 1 *i* ≤4)	134
Long-range NOEs (*i*, *i* ≥5)	734
Torsion angles	297 (90 *F*, 90 *Y*, 117 *W*)
Backbone hydrogen bonds	132 (33 hydrogen bonds)
Disulfide bond	6 (Cys^54^--Cys^123^)

**Violations and energies**	
Maximum distance violation	0.18 Å
Maximum dihedral angle violation	4.9°
Number of distance violations >0.15 Å	7
Number of dihedral angle violations >5°	0
Mean AMBER energy	−4314.4 kcal mol^−1^
Mean constraint energy	5.2 kcal mol^−1^

**Deviations for idealized geometry**	
Bond lengths	96.8 × 10^−4^ ± 0.6 × 10^−4^ Å
Bond angles	1.89 ± 0.02°

**Root mean square deviations from mean structure**	
Backbone heavy atoms	1.52 ± 0.31 Å
Backbone heavy atoms (structured region)*^a^*	0.55 ± 0.12 Å
All heavy atoms	2.27 ± 0.30 Å
All heavy atoms (structured region)*^a^*	1.38 ± 0.18 Å

**Ramachandran plot (*F* and *Y* angle distribution (%))*^b^***	
Residues in favored regions	96.3 (3911/4060)
Residues in additional allowed regions	99.9 (4057/4060)

*^a^* Values for the regions adopting regular secondary structure.

*^b^* Determined using the program Molprobity.

### Overall Structure and Comparison with Mouse Apo-PD-1

The structure shows that hPD-1, comprising residues 16–127 of the mature polypeptide, consists of a two-layer β sandwich with the topology of IgSF domains (*i.e.* two β sheets (GFCC′ and ABED) stabilized by a disulfide bond (Cys^34^–Cys^103^; Fig. 1*B*). Following determination of our structure, the coordinates for an equivalent form of hPD-1 obtained crystallographically were deposited in the PDB (PDB accession number 3RRQ). The two structures exhibit a very high degree of similarity (Fig. 1*C*). Automated structure comparisons using DALI (52) identified antigen receptor variable domains and the extracellular V-set domain of CTLA-4 as the structures most similar to hPD-1, as expected (21). The only significant difference between hPD-1 and these receptors was the extra flexibility in the region flanked by the C′ and D β strands (Fig. 1*D*). Detailed comparisons of human and mouse apo-PD-1 (53) reveal that although, overall, they are very similar (root mean square differences <1.30 Å for 104 Cα atoms), there are two regions of significant local differences (Fig. 1*E*). First, Pro^110^ imposes a twist in the FG loop of hPD-1, allowing orthodox positioning of the BC loop, whereas in mPD-1 the BC loop is drawn toward the DE loop by a hydrophobic interaction involving Arg^83^ (DE loop) and Trp^39^ (BC loop). The second and most important region of difference is at the edge of the GFCC′/GFCC′C″ sheets where, for hPD-1, strand C″ is completely absent. Overall, the extracellular regions of human and mouse PD-1 are relatively highly conserved (∼65%). The region of the C′D loop is among the least conserved parts of the sequence (<50%; Fig. 1*F*), but the key difference is the substitution of Cys for Pro at position 63 of hPD-1, which shortens the C′ strand by one residue and redirects the next eight residues away from strand C′, producing a highly flexible loop (Fig. 1*A*).

### Structural Basis of PD-L1 and PD-L2 Recognition

**FIGURE 2.**
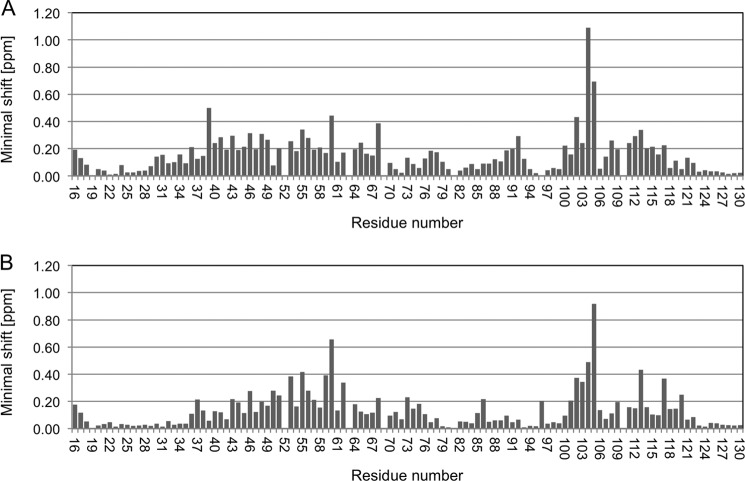
**Minimal backbone chemical shift (^15^N, ^13^C′, and ^1^H^N^) change values observed for hPD-1 on hPD-L1 (*A*) or hPD-L2 (*B*) binding.** The values were obtained by comparison of three-dimensional TROSY-HNCO spectra of the samples consisting of the free or hPD-L1/hPD-L2-bound ^2^H/^13^C/^15^N-labeled hPD-1.

**FIGURE 3.**
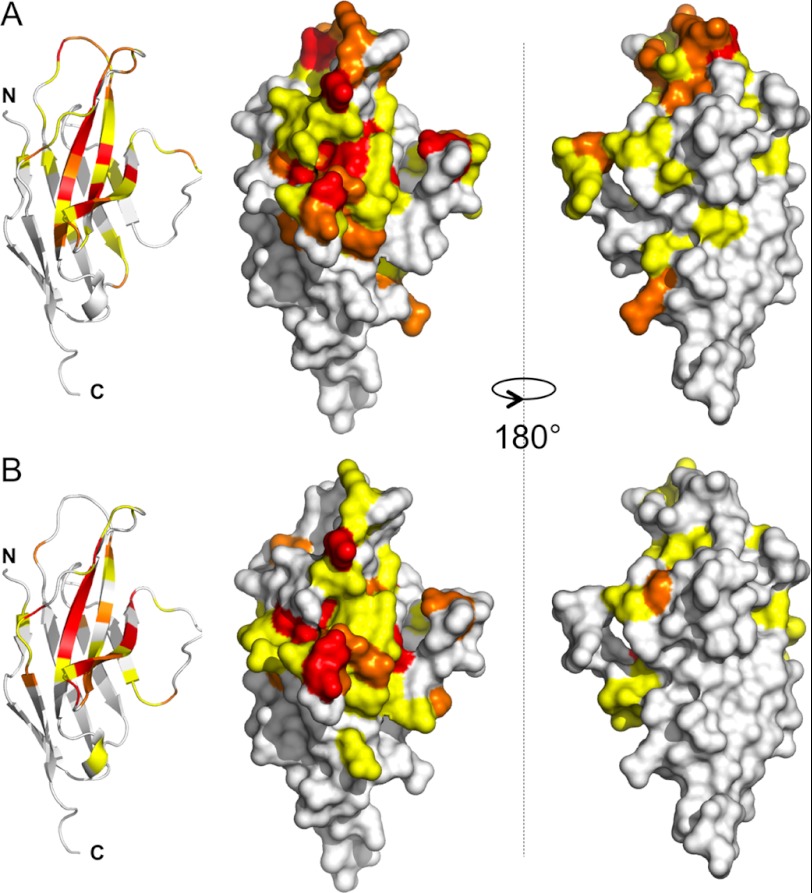
**Regions of hPD-1 affected by PD-L1/PD-L2 binding.** *A*, schematic and surface views of hPD-1 in which residues are *colored* according to the perturbation of their backbone (^15^N, ^13^C′, and ^1^H^N^) signals induced by hPD-L1 binding. Residues *highlighted* in *B* indicate the areas in hPD-1 affected by hPD-L2 binding. The *color scheme* used is relative for each complex (residues with minimal shift values lower than the S.D. value for the whole set are represented in *white*, residues with minimal shift values of >1 × S.D. are shown in *yellow*, residues with minimal shift values of >1.5 × S.D. are shown in *orange*, and residues with minimal shift values of >2 × S.D. are in *red*). Two views rotated by 180° are shown.

hPD-1·ligand complex formation was followed via perturbations of hPD-1 backbone NMR signals (^15^N, ^13^C′, and ^1^H^N^) induced by the ligands, hPD-L1 and hPD-L2 (Figs. 2 and 3). The changes were highly localized to a patch of residues on one face of hPD-1, with apparently no evidence of conformational changes being induced beyond this region. The addition of hPD-L1 significantly alters the positions of backbone signals for hPD-1 GFCC′ β sheet residues centered on Gln^55^ but also including Phe^43^, Asn^46^, Tyr^48^, and Arg^49^ (β-strand C); Ser^53^ (CC′ loop); Thr^56^ and Ala^60^ (strand C′); Asp^65^ and Gln^68^ (C′D loop); Arg^92^ (strand E); Thr^100^, Leu^102^, Cys^103^, Gly^104^, and Ala^105^ (strand F); Ala^112^ and Gln^113^ (FG loop); Ser^117^ (strand G); Thr^39^, Ser^40^, and Glu^41^ (BC loop); and Leu^108^ and Lys^111^ (FG loop; Fig. 3*A*). These residues presumably form part of an interaction surface, first identified in crystals of mPD-1 and hPD-L1 (24). Similar but not identical shifts appeared in the presence of hPD-L2, involving a subset of the Gln^55^-centered residues perturbed by hPD-L1 (*i.e.* Phe^43^, Asn^46^, Ser^53^, Thr^56^, Ala^60^, Gln^68^, Leu^102^, Cys^103^, Gly^104^, Ala^105^, Gln^113^, and Ser^117^), but also including Met^50^, Ser^51^, Asp^57^, Leu^59^, Phe^62^, Ser^73^, Phe^86^, and Ala^120^ of the GFCC′ β sheet and Ser^37^ of the BC loop (Figs. 2*B* and 3*B*). Thus, the hPD-L2 binding surface is centered on Gln^55^ of hPD-1 but with apparently smaller contributions from the hPD-1 BC and FG loops than for hPD-L1 binding. Overall, 22 of the 33 residues perturbed by either ligand in hPD-1 are conserved in mPD-1.

### Models of the Complexes

**FIGURE 4.**
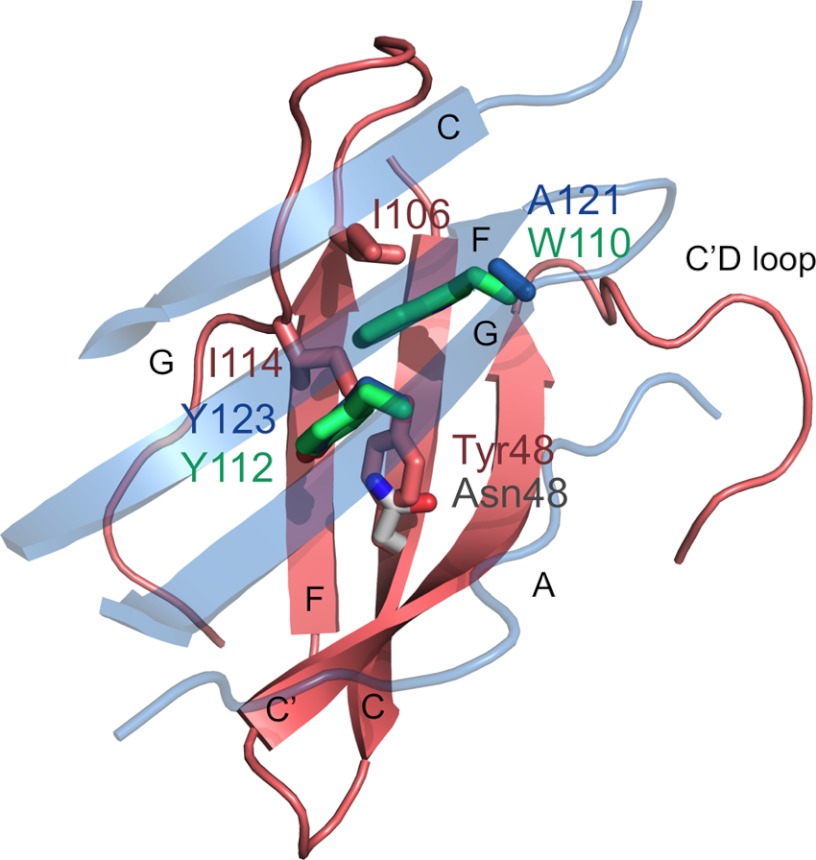
**Portions of modeled or actual complexes of hPD-1 (*red*) and mPD-1 (*gray*), complexed with hPD-L1 (*blue*) and mPD-L2 (*green*).** Only the GFCC′ strands and extended C′D loop of hPD-1 and the AGFC strands of hPD-L1 are shown. hPD-1 (*red*), mPD-1 (*gray*), hPD-L1 (*blue*), and mPD-L2 (*green*) residues for which the model or actual complexes appear to be inconsistent with the levels of perturbation of the hPD-1 backbone signals in the presence of the ligands are shown in *stick format* (see “Results” for details).

To help interpret these apparent differences in binding modes, models of hPD-1·hPD-L1 and hPD-1·mPD-L2 complexes were built by superimposing hPD-1 onto the solved mPD-1·hPD-L1 (PDB code 3BIK) and mPD-1·mPD-L2 (PDB code 3BP5) structures (24, 25). hPD-1 surfaces buried in the models exhibit close overlap with those perturbed in the NMR analysis of binding, as expected given the high degree of conservation of the GFCC′ sheet of PD-1 (data not shown) (53). However, there also appear to be significant discrepancies. First, in the modeled complexes, Tyr^48^ at the center of the binding region of hPD-1 (Asn in mPD-1) interacts with a tyrosine (Tyr^123^ in hPD-L1, Tyr^112^ in mPD-L2) conserved in the ligands of both species (Fig. 4), but this residue is perturbed only by hPD-L1 in the NMR analysis. The substitution of Tyr^48^ for Asn might otherwise have at least partly accounted for the weaker binding of the mouse proteins. Second, the C′ strand of hPD-1 is perturbed in the presence of both hPD-L1 and hPD-L2 but not contacted by either ligand in the modeled complexes. Flexibility or a conformational change in the C′-D loop of hPD-1 might account for this difference. Third, in the modeled hPD-1·mPD-L2 complex a conserved tryptophan (Trp^110^) in strand G (Ala in PD-L1) is well positioned to contact Ile^106^ and Ile^114^ of hPD-1 (Fig. 4), but neither Ile^106^ nor Ile^114^ are perturbed by the ligands. However, the minimal shift values for these residues might have been underestimated due to spectral overlap with signals from other residues. This contact would account for the stronger binding of hPD-1 to hPD-L2 *versus* hPD-L1 (discussed below). Mutation of Ile^106^ to Ala reduces ligand binding by 70–80%, and mutation of Ile^114^ to Ala completely abrogates it (53). mPD-L2 undergoes slight conformational rearrangements in the region of the start of the C′ strand and the BC loop when it binds mPD-1, whereas hPD-L1 binding is more “rigid body” in character (discussed in Ref. 25). These changes may be larger in hPD-L2 where the BC loop is not stabilized by disulfide bonding to the F strand (Cys^49^–Cys^106^). hPD-L2 binding may thus have substantial “induced fit” character, explaining its high affinity for PD-1 and the high enthalpy of the interaction (discussed below).

### Biophysical Basis of Ligand Binding

#### 

##### Protein Expression

Large amounts of soluble, histidine-tagged forms of human and mouse hPD-L1 and hPD-L2 were produced in CHO cells, using approaches described previously (27–29). Biotinylated forms of the proteins were also expressed transiently in HEK 293T cells, as also described previously (46). In reducing and non-reducing SDS-polyacrylamide gels, the proteins migrated as broad bands of 45–60 kDa, consistent with heavy glycosylation; on gel filtration, the proteins eluted at the positions expected for monomers, which was taken to indicate that they were correctly folded (data not shown). Human and mouse PD-1 were expressed stably in the form of thrombin-cleavable chimeras with human IgG Fc (designated PD-1Fc) and released with thrombin prior to use as analytes (47). Soluble forms of human and mouse B7-1 were prepared as described previously (29) (all construct details are shown in supplemental Table S1).

##### Affinity and Kinetic Measurements

**FIGURE 5.**
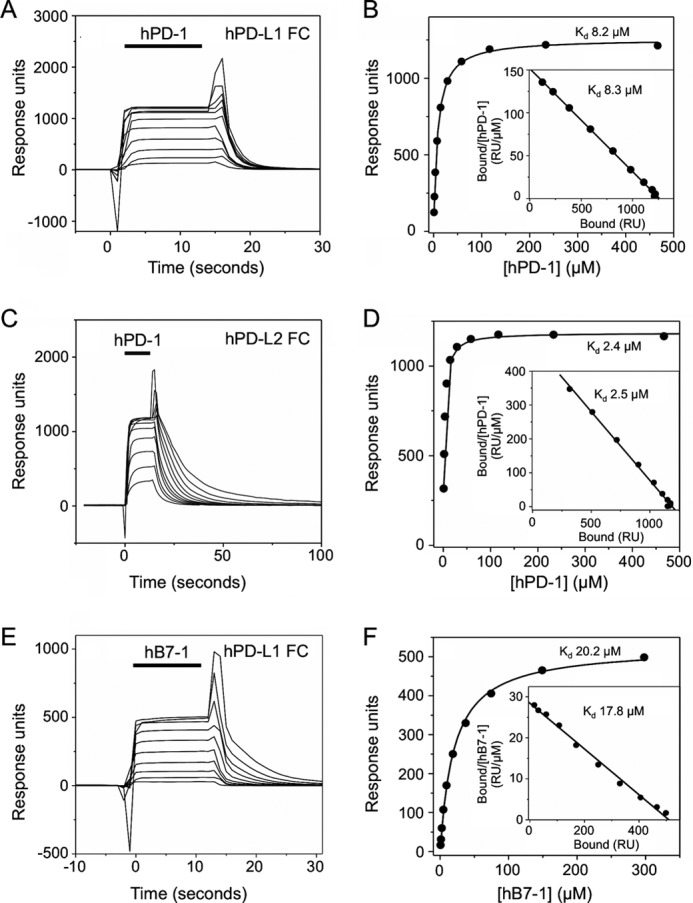
**Human PD-1/PD-1 ligand and hPD-L1/B7-1 interactions (equilibrium binding analyses).** *A*, hPD-1, at a range of concentrations (930 μm and 2-fold dilutions thereof), was injected at 20 μl/min sequentially (*solid bar*) through a flow cell containing ∼2000 RU of indirectly immobilized hPD-L1 at 37 °C. Background responses observed in a control flow cell containing immobilized hCD4 were subtracted from the total responses to give binding. *B*, nonlinear curve fitting of the untransformed data using a 1:1 Langmuir binding isotherm yielded a *K_d_* of 8.2 μm and a binding maximum of 1257 RU. A linear Scatchard plot of the hPD-1/hPD-L1 binding data (*inset*) yielded a similar *K_d_* of 8.3 μm. *C*, hPD-1, at a range of concentrations (930 μm and 2-fold dilutions thereof), was injected as in *A* through a flow cell containing ∼2000 RU of indirectly immobilized hPD-L2 at 37 °C. Background responses have been subtracted. *D*, nonlinear curve fitting of the untransformed data using a 1:1 Langmuir binding isotherm yielded a *K_d_* of 2.4 μm and a binding maximum of 1185 RU. A linear Scatchard plot of the hPD-1/hPD-L2 binding data (*inset*) yielded a similar *K_d_* of 2.5 μm. *E*, hB7-1, at a range of concentrations (596 μm and 2-fold dilutions thereof), was injected as in *A* through a flow cell containing ∼2000 RU of indirectly immobilized hPD-L1 at 37 °C. Background responses have been subtracted. *F*, nonlinear curve fitting of the untransformed data using a 1:1 Langmuir binding isotherm yielded a *K_d_* of 20.2 μm and a binding maximum of 525 RU. A linear Scatchard plot of the hB7-1/hPD-L1 binding data (*inset*) yielded a similar *K_d_* of 17.8 μm.

**FIGURE 6.**
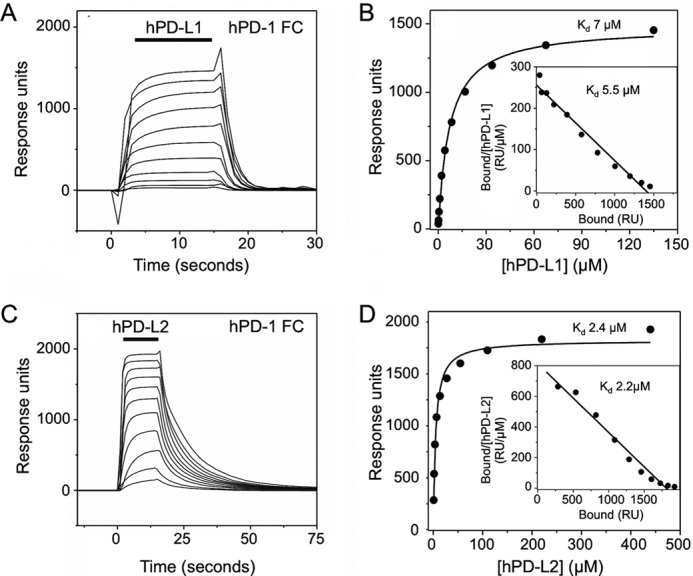
**hPD-1/hPD-L1 equilibrium binding analyses (reverse orientation).** *A*, hPD-L1, at a range of concentrations (135 μm and 2-fold dilutions thereof) was injected at 20 μl/min sequentially (*solid bar*) through a flow cell containing ∼4000 RU of directly immobilized hPD-1Fc at 37 °C. Background responses observed in a control flow cell containing immobilized hCD28Fc were subtracted from the total responses to give binding. *B*, nonlinear curve fitting of the untransformed data using a 1:1 Langmuir binding isotherm yielded a *K_d_* of 7.0 μm and a binding maximum of 1480 RU. A linear Scatchard plot of the hPD-1/hPD-L1 binding data (*inset*) yielded a similar *K_d_* of 5.5 μm. *C*, hPD-L2, at a range of concentrations (220 μm and 2-fold dilutions thereof), was injected as in *A* through a flow cell containing ∼5000 RU of directly immobilized hPD-1Fc at 37 °C. Background responses have been subtracted. *D*, nonlinear curve fitting of the untransformed data using a 1:1 Langmuir binding isotherm yielded a *K_d_* of 2.4 μm and a binding maximum of 1822 RU. A linear Scatchard plot of the hPD-1/hPD-L2 binding data (*inset*) yielded a similar *K_d_* of 2.2 μm.

**FIGURE 7.**
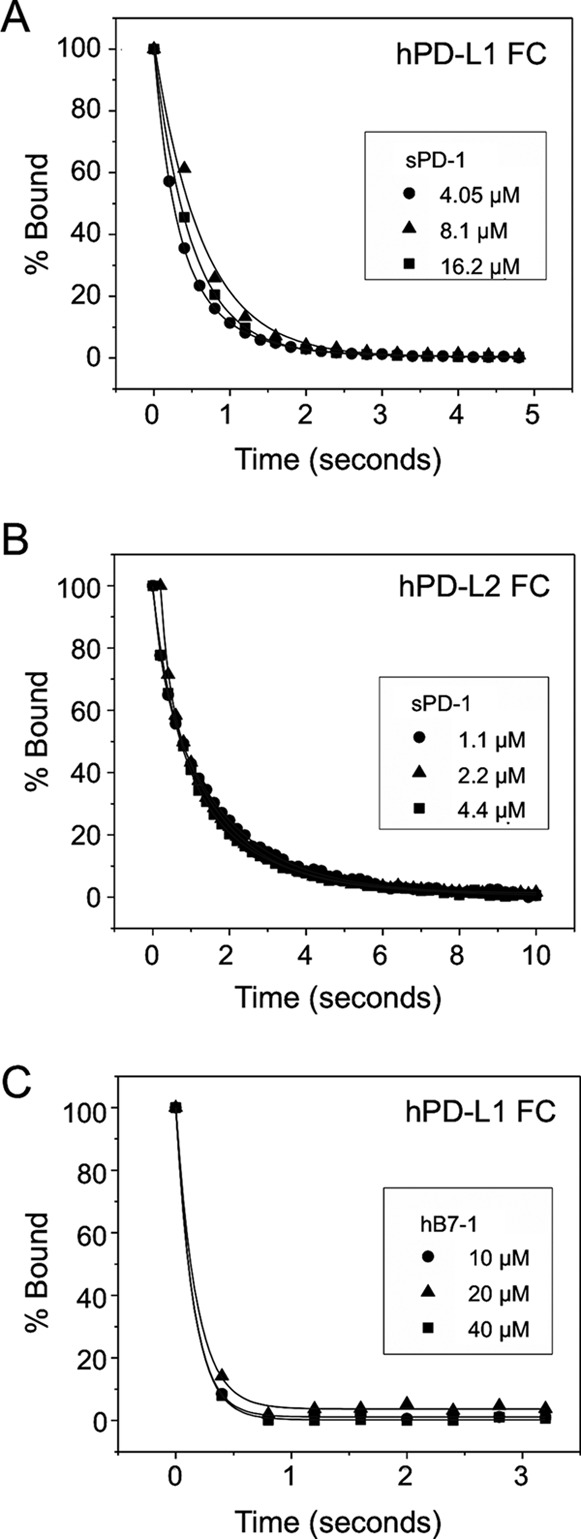
**Kinetic analyses.** *A*, hPD-1, at concentrations of 4.05, 8.1, and 16.2 μm, was injected at 100 μl/min at 37 °C over ∼250 RU of indirectly immobilized hPD-L1 and allowed to dissociate at the end of each injection. Data were recorded at the maximal collection rate (10 Hz) until the response had returned to base line. Responses in a control flow cell were subtracted, and the remaining binding was plotted as a percentage of initial binding. The data are fitted with single exponential decay curves, giving a *k*_off_ value of 1.97 ± 0.19 s^−1^ (mean ± S.D., *n* = 9). *B*, dissociation of hPD-1 from hPD-L2 at 37 °C. hPD-1 (1.1, 2.2, and 4.4 μm) was injected over ∼100 RU of indirectly immobilized hPD-L2 at 100 μl/min. The data are fitted with single exponential decay curves, giving a *k*_off_ value of 0.71 ± 0.07 s^−1^ (mean ± S.D., *n* = 9). *C*, dissociation of hB7-1 from hPD-L1 at 37 °C. hB7-1 (10, 20, and 40 μm) was injected over ∼350 RU of indirectly immobilized hPD-L1 at 100 μl/min. The data are fitted with single exponential decay curves, giving a *k*_off_ value of 6.44 ± 0.38 s^−1^ (mean ± S.D., *n* = 9).

PD-1/ligand interactions were characterized using SPR-based assays at 37 °C. For equilibrium analysis of affinity, increasing amounts of hPD-1 were injected over immobilized biotinylated hPD-L1, hPD-L2, and sCD4 (used as a negative control). Binding reached equilibrium rapidly (>95% binding within 1–3 s), and during the wash phase, the base-line signal recovered quickly (within 5–15 s), reflecting very fast kinetics. Representative sensorgrams are shown in Fig. 5, *A* and *C*. Plots of specific binding *versus* concentration indicated that binding was saturable (Fig. 5, *B* and *D*). The good fit of the data to 1:1 Langmuir binding isotherms (Fig. 5, *B* and *D*) and the linear Scatchard plots (Fig. 5, *B* and D, *insets*) were consistent with a simple 1:1 Langmuir binding model. *K_d_* values of 8.2 ± 0.1 μm (mean ± S.D., *n* = 2) and 2.3 ± 0.1 μm (*n* = 2), respectively, were obtained for the binding of hPD-1 to hPD-L1 and hPD-L2 (Table 2). Measurements of these affinities in the opposite orientation (*i.e.* with hPD-1 immobilized and the ligands used as analytes) were in good agreement (Fig. 6 and Table 2). The binding of hB7-1 to hPD-L1 (*K_d_* ∼18.8 ± 3.8 μm (*n* = 6); Fig. 5, *E* and *F*) was substantially weaker than reported previously (∼1.7 μm) (23) for hB7-1 injected over immobilized hPD-L1. In the opposite orientation, a slightly higher *K_d_* of 35.4 ± 4.4 μm was obtained (Table 2). Kinetic analysis revealed that all affinity differences were almost entirely attributable to off-rate variation (Fig. 7 and Table 2).

**TABLE 2:** **Affinity and kinetic parameters for PD-1 binding to PD-L1 and PD-L2** Interactions of human and mouse PD-1 with PD-L1 or PD-L2 were characterized at 37 °C using SPR-based assays as implemented by Biacore^TM^.

	Interaction								
	hPD-1/hPD-L1		hPD-1/hPD-L2		hPD-L1/hB7-1		mPD-1/mPD-L1	mPD-1/mPD-L2	mPD-L1/mB7-1
Analyte	hPD-1	hPD-L1	hPD-1	hPD-L2	hPD-L1	hB7-1	mPD-1	mPD-1	mB7-1
*n*	2	6	2	6	4	6	8	8	6
*K_d_* (μm)*^a^*	8.2 ± 0.1	7.5 ± 2.2	2.3 ± 0.1	2.2 ± 0.2	35.4 ± 4.4	18.8 ± 3.8	29.8 ± 6.8	38.4 ± 7.1	79.3 ± 3.6
*k*_on_*^b^* (m^−1^ s^−1^)	1.84 × 10^5^		2.5 × 10^5^		3.16 × 10^5^		ND*^c^*	ND	ND
*k*_off_*^d^* (s^−1^)	1.44 ± 0.21		0.55 ± 0.05		5.94 ± 0.12		ND	ND	ND

*^a^* Means and S.D. are shown; *K_d_* values were obtained by non-linear fitting of 1:1 Langmuir binding isotherms.

*^b^* Each *k*_on_ was calculated from the equation *k*_on_ = *k*_off_/*K_d_*.

*^c^* ND, not determined.

*^d^ k*_off_ (*k*_off_ = mean ± S.D.) was calculated via the fitting of global 1:1 binding models to the data.

**FIGURE 8.**
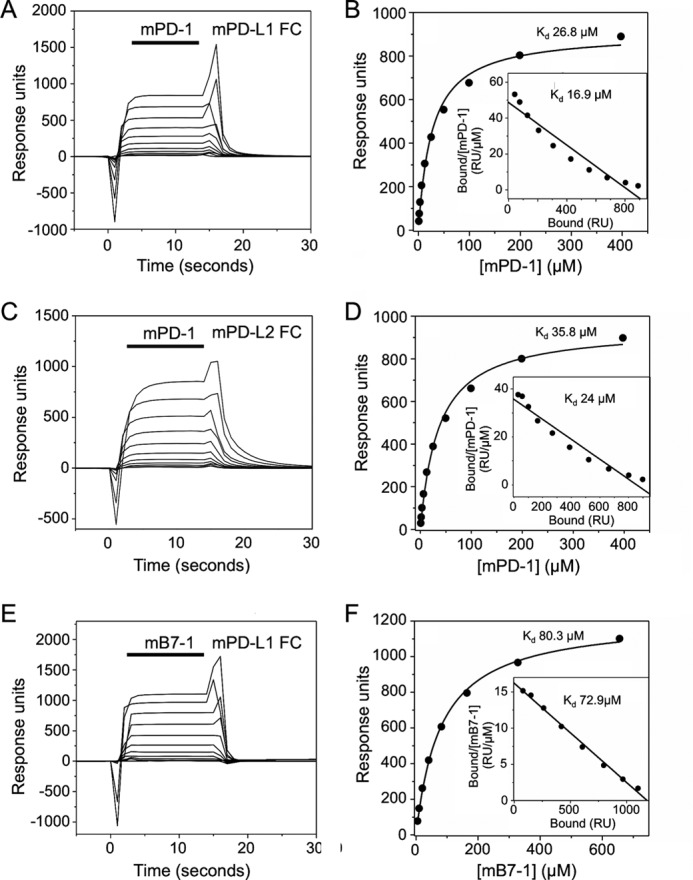
**Murine PD-1/PD-L1 interactions (equilibrium binding analyses).** *A*, mPD-1, at a range of concentrations (398 μm and 2-fold dilutions thereof) was injected at 20 μl/min sequentially (*solid bar*) through a flow cell containing ∼2000 RU of indirectly immobilized mPD-L1 at 37 °C. Background responses observed in a control flow cell containing immobilized hCD4 were subtracted from the total responses to give binding. *B*, nonlinear curve fitting of the untransformed data using a 1:1 Langmuir binding isotherm yielded a *K_d_* of 26.8 μm and a binding maximum of 908 RU. A Scatchard plot of the mPD-1/mPD-L1 binding data (*inset*) yielded a *K_d_* of 16.9 μm. *C*, mPD-1, at a range of concentrations (398 μm and 2-fold dilutions thereof) was injected as in *A* through a flow cell containing ∼2000 RU of indirectly immobilized mPD-L2 at 37 °C. Background responses have been subtracted. *D*, nonlinear curve fitting of the untransformed data using a 1:1 Langmuir binding isotherm yielded a *K_d_* of 35.8 μm and a binding maximum of 946 RU. A Scatchard plot of the mPD-1/mPD-L2 binding data (*inset*) yielded a *K_d_* of 24 μm. *E*, mB7-1, at a range of concentrations (655 μm and 2-fold dilutions thereof) was injected as in *A* through a flow cell containing ∼2000 RU of indirectly immobilized mPD-L1 at 37 °C. Background responses have been subtracted. *F*, nonlinear curve fitting of the untransformed data using a 1:1 Langmuir binding isotherm yielded a *K_d_* of 80.3 μm and a binding maximum of 1215 RU. A Scatchard plot of the mB7-1/mPD-L1 binding data (*inset*) yielded a similar *K_d_* of 72.9 μm.

The murine interactions were considerably weaker than the analogous human interactions. Moreover, the mPD-1/ligand affinities were the same, within error (*K_d_* 29.8 ± 6.8 (*n* = 8) and 38.4 ± 7.1 μm (*n* = 8) for mPD-1 binding to mPD-L1 and mPD-L2, respectively), in marked contrast to the interactions of hPD-1 (Fig. 8, *A–D*). The affinity of mB7-1 for mPD-L1 was very low, at 80.3 ± 6.8 μm (*n* = 6; Fig. 8, *E* and *F*, and Table 2).

##### Thermodynamic Measurements

**FIGURE 9.**
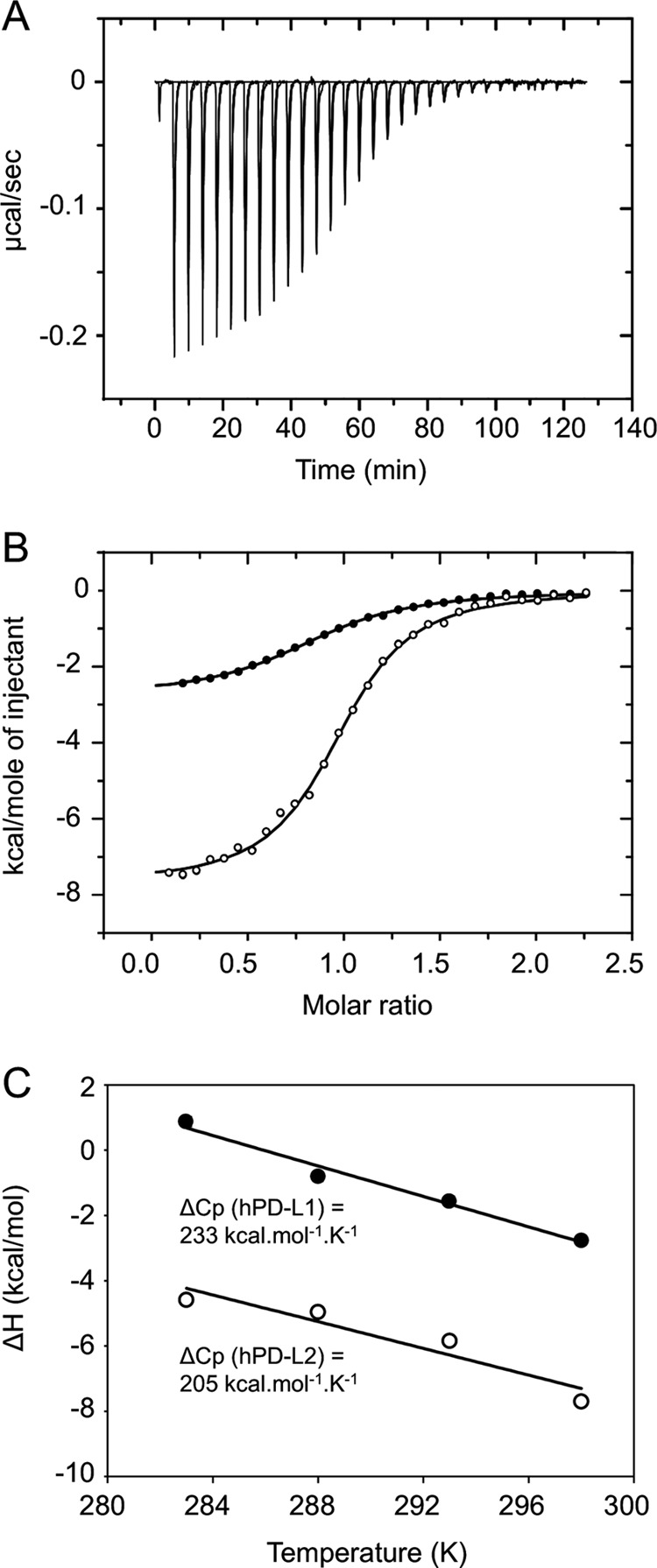
**ITC measurements of hPD-1 binding to PD-L1 and PD-L2.** *A*, example of raw data for titration of hPD-L2 at 0.2 mm into an isothermal calorimetry cell containing a 0.02 mm solution of hPD-1Fc at pH 7.4 and 25 °C in a buffer containing 150 mm NaCl. Similar titrations were undertaken at various temperatures, the results of which are summarized in Table 3. *B*, plots of heat-released *versus* molar ratio for the interactions of hPD-L1 (*closed circles*) and hPD-L2 (*open circles*) with hPD-1. *C*, plots of observed enthalpy *versus* temperature for the interactions of hPD-L1 (*closed circles*) and hPD-L2 (*open circles*) with hPD-1. The slopes of these plots give the change in heat capacity (Δ*Cp*) upon binding of hPD-L1 and hPD-L2 to hPD-1, the values of which are −233 and −205 cal mol^−1^ K^−1^, respectively.

For thermodynamic analysis, the soluble forms of PD-L1 and PD-L2 were mixed with hPD-1 in the form of uncleaved Fc fusion protein. Representative data for the isothermal calorimetric titration of the hPD-1Fc dimer with hPD-L1 and hPD-L2 at 25 °C are shown in Fig. 9, *A* and *B*; titrations performed at other temperatures gave similar quality data (Table 3) (data not shown). The heats of interaction were not very large, but isotherms could be readily fitted to the data. The binding stoichiometry for both ligands is 1:1, in accordance with the finding that human PD-1 is monomeric in solution and at the cell surface (53). At the temperatures investigated (10–25 °C), the affinity of hPD-L2 for hPD-1Fc was ∼5–8-fold higher than that of hPD-L1 for hPD-1Fc, in good agreement with the ratio obtained by SPR analysis (Tables 2 and 3). However, the SPR-derived affinities were somewhat lower than those measured by ITC, as observed elsewhere (21).

**TABLE 3:** **Thermodynamic properties of hPD-1 binding to hPD-L1 and hPD-L2** Interactions of PD-1 with PD-L1 or PD-L2 were characterized at a range of temperatures from 10 to 25 °C by quantitative ITC analysis.

	Binding parameters					
	*n**^a^*	*K*	*K_d_**^a^*	Δ*H**^a^*	−*T*·Δ*S**^b^*	Δ*G*
		*10^5^m*^−*1*^	μ*m*	*kcal mol*^−*1*^	*kcal mol*^−*1*^	*kcal mol*^−*1*^
**hPD-1 binding to hPD-L1**						
pH 6.0	0.904 ± 0.0098	6.28 ± 0.47	1.6	−2.23 ± 0.033	−5.66	−7.9
pH 7.4	0.865 ± 0.015	4.58 ± 0.49	2.2	−2.88 ± 0.068	−4.83	−7.7
pH 8.0	0.906 ± 0.018	3.29 ± 0.34	3.04	−2.69 ± 0.077	−4.83	−7.5
10 °C	1.23 ± 0.032	2.96 ± 0.65	3.38	0.87 ± 0.037	−7.95	−7.5
15 °C	0.602 ± 0.014	5.76 ± 1.80	1.74	−0.81 ± 0.078	−6.8	−7.8
20 °C	0.882 ± 0.014	5.11 ±0.48	1.96	−1.56 ± 0.035	−6.09	−7.8
25 °C	0.878 ± 0.057	5.41 ± 0.21	1.85	−2.77 ± 0.026	−5.07	−7.8
50 mm NaCl	0.842 ± 0.0073	6.15- ± 0.34	1.63	−3.24 ± 0.038	−4.65	−7.9
500 mm NaCl	0.902 ± 0.017	5.15 ± 0.61	1.94	−2.21 ± 0.058	−5.57	−7.8

**hPD-1 binding to hPD-L2**						
pH 6.0	1.08 ± 0.0055	20.2 ± 0.97	0.5	−6.18 ± 0.043	−2.42	−8.6
pH 7.4	1.11 ± 0.0075	22.2 ± 1.50	0.45	−6.50 ± 0.059	−2.15	−8.66
pH 8.0	1.04 ± 0.006	17.8 ± 0.90	0.56	−7.24 ± 0.057	−1.29	−8.53
10 °C	0.982 ± 0.012	23.7 ± 2.60	0.42	−4.57 ± 0.081	−3.71	−8.5
15 °C	0.988 ± 0.015	37.7 ± 6.80	0.27	−4.96 ± 0.10	−3.72	−8.8
20 °C	1.09 ± 0.018	29.5 ± 5.50	0.34	−5.84 ± 0.14	−2.84	−8.7
25 °C	0.979 ± 0.0063	26.3 ± 1.70	0.38	−7.70 ± 0.067	−1.06	−8.8
50 mm NaCl	1.01 ± 0.0056	20.8 ± 1.10	0.48	−7.57 ± 0.057	−1.05	−8.6
500 mm NaCl	0.985 ± 0.0068	21.7 ± 1.40	0.46	−7.35 ± 0.068	−1.29	−8.6

*^a^* The values for *K_d_*, stoichiometry, and Δ*H*, obtained by fitting a single binding site model to the ITC data, are shown with S.E. values.

*^b^* The Δ*S* value was calculated using the equation, Δ*G* = −*RT* × ln(*K_a_*) = Δ*H*_obs_ − *T*Δ*S*.

Thermodynamic parameterization (Table 3) reveals that the hPD-1/ligand interactions are subtly different. Under all conditions, both interactions have favorable Δ*H*_obs_ and *T*Δ*S*, but hPD-1/hPD-L1 binding is entropically driven, whereas the hPD-1/hPD-L2 interaction has a large enthalpic term. The binding differences do not seem to be based on charge or protonation effects because the data are largely invariant at the different pH values and salt concentrations. The differences are not manifested in the temperature dependence of the thermodynamic parameters either. Δ*H*_obs_ for the association of hPD-1 with hPD-L1 and hPD-L2 declines linearly from +0.8 kcal mol^−1^ at 15 °C to −2.77 kcal mol^−1^ at 25 °C and from −4.6 kcal mol^−1^ to −7.7 kcal mol^−1^, respectively (Fig. 9*C*), yielding very similar Δ*Cp* terms of −233 and −205 cal mol^−1^ K^−1^, respectively. Because Δ*Cp* is usually dominated by solvent effects, it implies that the hydrophobic surface areas buried in forming the two complexes are very similar. Overall, the interaction of hPD-1 with its ligands is robust with respect to possible fluctuations in extracellular conditions. The lack of effects of temperature, pH, and salt conditions suggests that the differences in affinity and thermodynamic parameters are most likely the result of minor changes in the formation of non-covalent interactions in the binding sites.

### Simulations of PD-1/Ligand and PD-L1/B7-1 Interactions

#### 

##### Molecule Expression Levels

**FIGURE 10.**
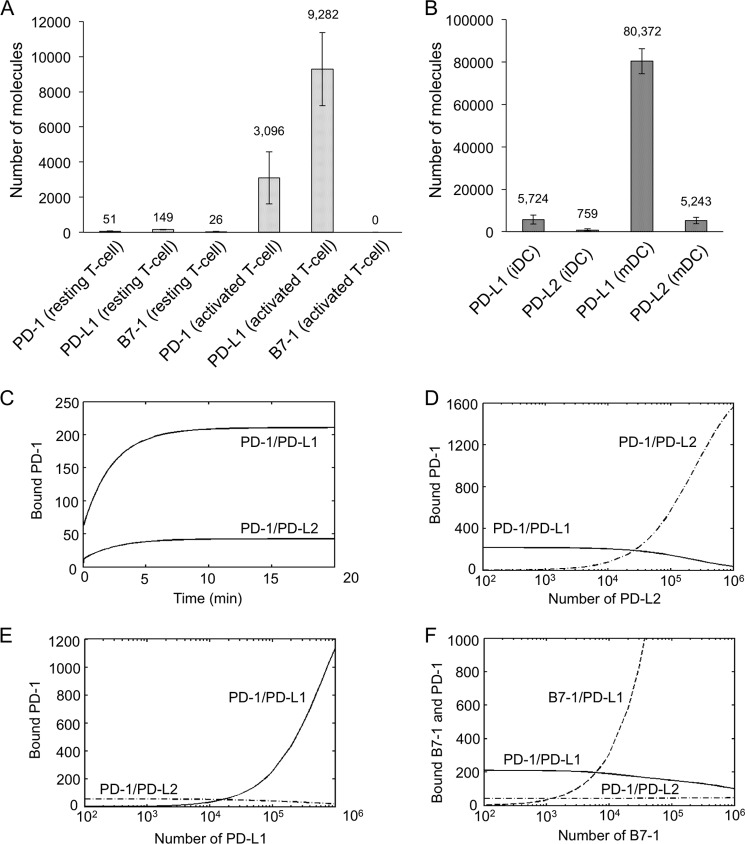
**Simulations of human PD-1/ligand and PD-L1/B7-1 interactions based on affinity and expression data.** *A*, the expression levels of human PD-1, PD-L1, and B7-1 on resting and activated human T cells. Peripheral blood mononuclear cells were activated with PHA (50 μg/ml) for 2 days. Cells were stained with PE-conjugated mAbs for each protein and analyzed by flow cytometry. QuantiBRITE PE beads were analyzed alongside the stained peripheral blood mononuclear cell samples. The experiments were done in duplicate. The average of two sets of data is shown; the *error bars* indicate S.D. *B*, expression levels for PD-L1 and PD-L2 on immature DCs and mature DCs. DCs were derived by using GM-CSF (50 ng/ml) and IL-4 (50 ng/ml) for 6 days. CD14^+^ monocytes were initially isolated from human peripheral blood mononuclear cells using CD14 MACs beads (Miltenyi Biotec). Immature DCs were then stimulated with LPS (1 μg/ml) for 24 h to obtain mature DCs. Cells were stained with PE-conjugated mAbs for each protein and analyzed by flow cytometry. The experiments were done in duplicate. The average of two sets of data are shown; *error bars* indicate S.D. *C–F*, simulations of molecular complex formation at the synaptic interface between an activated T cell and a mature DC. *C*, numbers of bound PD-1 molecules over time. *D* and *E*, number of bound PD-1 molecules at steady state as a function of varying the number of PD-L2 or PD-L1 molecules on the DC. *F*, number of bound PD-1 and B7-1 molecules at steady state as a function of varying the number of B7-1 molecules on the T cell.

In order to simulate complex formation by human PD-1 at cellular synapses, we obtained quantitative expression data for each of the proteins using flow cytometry. Resting and phytohemagglutinin-activated CD3^+^ human T cells were stained with phycoerythrin (PE)-conjugated anti-PD-1, anti-PD-L1, anti-PD-L2, and anti-B7-1 antibodies alongside beads conjugated with known different amounts of PE. hPD-1 and hPD-L1 were undetectable on resting T cells. Following activation, however, these proteins reached expression levels of ∼3000/cell and ∼9000/cell, respectively, consistent with previous findings (54, 55) (Fig. 10*A*). B7-1 was undetectable under either condition, however. PD-L1 and PD-L2 were expressed by *in vitro* prepared (50) immature and mature human DCs at levels of ∼5000 and ∼700 copies/cell (immature DCs) and ∼80,000 and ∼5000 copies/cell (mature DCs), respectively (Fig. 10*B*). The numbers of CD28, CTLA-4, B7-1, and B7-2 molecules used in the simulations were based on previous estimates (50).

##### Simulations

**FIGURE 11.**
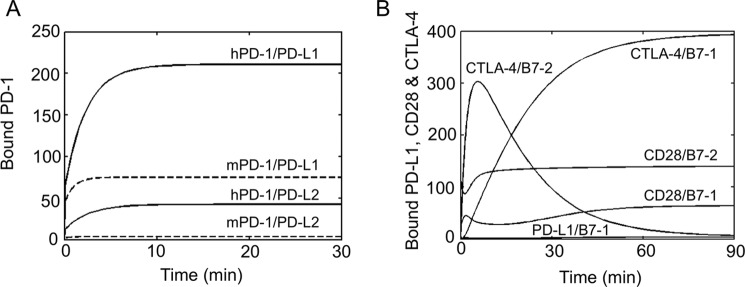
**Simulations of murine PD-1/ligand interactions and effects of PD-L1/B7-1 interactions on CD28 and CTLA-4 ligation.** *A*, number of bound PD-1 molecules over time. The simulations are based on human *K_d_* values (*solid lines*) and on mouse *K_d_* values (*dashed lines*) obtained in the present study. The expression values and off-rates obtained for human PD-1 and its ligands are used in both simulations because the corresponding mouse data are not available. *B*, number of bound PD-L1, CD28, and CTLA-4 as a function of time. The effect of PD-L1 on the CD28 and CTLA-4 ligation to their B7 ligands was simulated by incorporating the interaction between PD-L1 and B7-1 in our previous model of the costimulatory system (50). Because PD-L1 only binds to a few B7-1 molecules, PD-L1 expression barely affects CD28 and CTLA-4 ligation.

Complex formation was simulated using a system of nonlinear ordinary differential equations, incorporating the stoichiometric, affinity, and expression data, as described previously (Table 4) (50) (see supplemental Experimental Procedures for details). Briefly, the three-dimensional *k*_on_ values were converted to two-dimensional on-rates using the methods of Bell (56). Simulations begin at the time an activated T cell forms a synapse with a mature DC because human PD-1 and PD-L1 were undetectable on naive cells. The cell surfaces are divided into the synapse (c-SMAC) and the region outside the synapse, and freely diffusing unbound mobile molecules are recruited to the synapse by ligation. In simulations of human PD-1/ligand interactions as a function of time (Fig. 10*C*), human PD-1 accumulation in the synapse reaches a steady state within 15 min. Although human PD-1 binds PD-L2 with a ∼3.5-fold higher affinity than it binds PD-L1, it forms 5-fold fewer PD-1·PD-L2 than PD-1·PD-L1 complexes at steady state due to the 15-fold lower expression of PD-L2 *versus* PD-L1. Varying the expression level of PD-L2 reveals that the human PD-1/PD-L1 interaction is only sensitive to increasing PD-L2 levels at very high levels of PD-L2 (>5 × 10^4^; Fig. 10*D*). Conversely, human PD-1 engagement by PD-L2 is largely insensitive to PD-L1 at all levels of PD-L1 expression (Fig. 10*E*). The relatively low level of human PD-1 engagement by PD-L2 is therefore not due to competition with PD-L1 at physiological expression levels. Simulations incorporating the *K_d_* values for the murine interactions (Table 2), using the human expression data, result in ∼3-fold and ∼20-fold reductions in mouse PD-1·PD-L1 and PD-1·PD-L2 complex formation, respectively (Fig. 11*A*), due to the weaker affinities.

**TABLE 4:** **Parameter values used for the computational simulations**

Name	Definition	Value	Reference/Source
*a*_Tcell_	Area of an activated T cell	452 μm^2^	Ref. 1
*a*_DC_	Area of a dendritic cell	1256 μm	Ref. 1
*a*_syn_	Area of synapse (c-SMAC)	12.6 μm^2^	Ref. 1
γ_DC_	Rate constant for molecules on DC diffusing into synapse	2.7 × 10^−4^ s^−1^	Ref. 1
κ_DC_	Rate constant for molecules on DC diffusing out from synapse	2.7 × 10^−2^ s^−1^	Ref. 1
γ_T_	Rate constant for molecules on T cells diffusing into synapse	1.0 × 10^−3^ s^−1^	Ref. 1
κ_T_	Rate constant for molecules on T cells diffusing out from synapse	3.6 × 10^−2^ s^−1^	Ref. 1
*m*	Fraction of mobile cell surface molecules	0.6*^a^*	Ref. 3
α_1_	Association of PD-1 and PD-L1	0.10 μm^2^ s^−1^	This work
α_2_	Association of PD-1 and PD-L2	0.14 μm^2^ s^−1^	This work
α_3_	Association of B7-1 and PD-L1	0.17 μm^2^ s^−1^	This work
α_4_	Association of B7-1 monomers	0.03 μm^2^ s^−1^	Ref. 4
α_33_	Bivalent association of B7-1 and PD-L1	0.17 μm^2^ s^−1^	This work
δ_1_	Dissociation of PD1·PDL1 complexes	1.44 s^−1^	This work
δ_2_	Dissociation of PD1·PDL2 complexes	0.55 s^−1^	This work
δ_3_	Dissociation of B71·PDL1 complexes	5.94 s^−1^	This work
δ_4_	Dissociation of B7-1 dimer	1 s^−1^	Ref. 4
δ_33_	Dissociation of bivalent B7-1·PD-L1 complexes	5.94 s^−1^	This work

*^a^* The motilities of the cell surface molecules were assumed to be the same as the ones observed for CD2 (50).

Although we were unable to detect B7-1 expression on activated T cells, others have (57–59). To explore the likelihood that B7-1 expression on T cells affects PD-1 engagement, we estimated the steady state levels of bound PD-1 and B7-1 molecules as a function of varying B7-1 expression. B7-1 only began to affect PD-1·PD-L1 complex formation when expressed at >5000 molecules/cell (Fig. 10*F*). It therefore seems unlikely that B7-1 would have any appreciable effect on PD-1 ligation because even mature DCs do not express more than 4000 B7-1 molecules/cell (50).

The *K_d_* values obtained in this study are 10–16-fold larger than those obtained previously (23). In simulations of B7-1/PD-L1 binding at the older, higher affinities, the numbers of bound PD-1 and B7-1 molecules at steady state increase in direct proportion to the respective affinity differences (data not shown). The implication is that the effects of PD-L1/B7-1 binding on the competing interactions of each of these proteins would have been significant if the previous measurements of these affinities were reliable. However, using the *K_d_* values obtained in the present study for the B7-1 and PD-L1 interaction, the inclusion of PD-L1 in our previous model (50) barely affects the ligation of CD28 and CTLA-4 (Fig. 11*B*).

## DISCUSSION

PD-1 plays an important role in down-regulating immune responses, reportedly via a number of different mechanisms after binding its ligands PD-L1 and PD-L2. Previous studies revealed that PD-L1 and PD-L2 have similar structures but very different expression patterns and expression kinetics (15). The unexpected finding that PD-L1 also binds B7-1 further complicates matters (22, 23). A detailed understanding of the structures and interactions of PD-1 should aid in rationalizing this complexity.

Our NMR-derived structure of human PD-1 is most similar to antigen receptor domains, consistent with a shared evolutionary origin (21). Human PD-1 is, however, surprisingly different from its murine ortholog. Whereas mouse PD-1 has a “conventional” IgSF V-set domain, the human receptor lacks a C″ strand, and instead the C′ and D strands are connected by a relatively long and flexible loop. Moreover, the BC loop is not stabilized by disulfide bonding to the F strand of the ligand binding β sheet. These interspecies differences are apparently responsible for the surprising differences in the affinities of human and mouse PD-1 for their ligands (discussed below) because mouse PD-L1 and PD-L2 have the same affinities for hPD-1 as their human counterparts (data not shown). Although human PD-1 is relatively flexible, this does not present a large barrier to ligand binding because the overall binding entropies are favorable. Perturbations of hPD-1 backbone NMR signals in the presence of its ligands, combined with thermodynamic analysis of binding, revealed that the ligands bind in apparently different ways to the same site on hPD-1, with PD-L2 appearing to form a smaller interface with possibly better geometric complementarity than PD-L1, aided perhaps by local conformational rearrangements. Another explanation for the enthalpic nature of this interaction is that during hPD-1 and PD-L2 binding, an additional water molecule(s) is incorporated. This would have a negative Δ*H* effect due to additional hydrogen bonding but at an entropic cost. The Δ*Cp* values for human PD-1/PD-L1 and PD-1/PD-L2 binding were very similar, consistent with the involvement of large hydrophobic areas (∼1000 Å^2^) in the binding of both ligands. However, neither ligand bound human PD-1 in a way that was fully explained by the crystal structures of the mouse PD-1·ligand complexes, although the binding surface, defined by the ligand-induced perturbations of the human PD-1 backbone residues, is relatively highly conserved (*i.e.* 22 of 33 residues). Overall, the divergence of the human and mouse structures seems only to have been constrained by the need to retain the ligand-binding surface.

Perhaps the most striking finding of the present study is that the interactions of this inhibitory receptor are relatively weak and much weaker than those of the other key inhibitory protein expressed by T cells, CTLA-4. Interactions within the CD28/CTLA-4 system differ in strength due to affinity differences and stoichiometric effects; the affinities vary by ∼2 orders of magnitude from the strongest (CTLA-4·B7-1) to the weakest (CD28·B7-2), with the bivalency of CTLA-4 and B7-1 further accentuating these differences (by ∼2 more orders of magnitude (28)), such that the half-lives of inhibitory (CTLA-4·B7-1) and activating (CD28·B7-2) complexes may differ >10,000-fold. For the human PD-1 system, such large differences are not possible; the affinities differ only 3–4-fold, and the proteins are all monovalent (53). Thus, the half-lives of human PD-1·PD-L1 (*K_d_* ∼8 μm) and PD-1·PD-L2 (*K_d_* ∼2 μm) signaling complexes will probably be 1000–5000-fold shorter than that of CTLA-4·B7-1 complexes. Murine PD-1·ligand complexes (30–35 μm) will probably be even shorter lived. The very stable CTLA-4·B7-1 complexes formed after T cell activation were thought to be required to turn off activating signals delivered by CD28 (28, 60). However, it now seems that very stable complexes are not a prerequisite for potent inhibitory signaling.

PD-1 engagement is more effective than CTLA-4 ligation in suppressing gene transcription induced by CD3/CD28-generated signals (61), also suggesting that PD-1 and CTLA-4 block T cell activation in different ways, given that PD-1 also relies on much weaker interactions. PD-1 and CTLA-4 both block Akt activation, albeit using distinct mechanisms (61); PD-1 inhibits Akt by blocking PI3K activation, whereas CTLA-4 uses PP2A to inhibit Akt. CTLA-4 engagement also disrupts the recruitment of ZAP70 to microclusters, reversing the “stop” signal induced by TCR signaling, thereby inhibiting activation (62). PD-1 is also proposed to disrupt the stop signal via the recruitment of the SHP-2 phosphatase to microclusters (14). But it is possible that CTLA-4 is not a conventional signaling receptor. Consistent with its formidable binding properties, it is suggested that CTLA-4 exerts its inhibitory effect on CD28 signaling by depleting their mutual ligands B7-1 and B7-2 from apposing cells via *trans*-endocytosis (63). The very much weaker interactions of PD-1 probably make it incapable of such effects, which would in any case only limit its inhibitory potential because it does not share ligands with activating receptors, unlike CTLA-4. The cytoplasmic domains of CTLA-4 and CD28 bind to a remarkably similar spectrum of Src homology 2 domains, suggesting that at the signaling level, CTLA-4 might not be any more inhibitory than CD28. PD-1, however, binds an entirely different set of Src homology 2 domains, including SHP-2, as expected for an inhibitory as opposed to an activating receptor.^5^ It might be that, in general, the binding affinities of conventional inhibitory receptors are not substantially different from those of activating receptors.

Once formed, human PD-1·PD-L2 complexes will probably be ∼3-fold more stable than PD-1·PD-L1 complexes. This could lead to differential phosphorylation of the tyrosine residues in the cytoplasmic ITIM and ITSM motifs of PD-1, resulting in qualitatively different signals in response to each of the ligands. Because the expression of PD-L2 is thought to be largely restricted to “professional” antigen-presenting cells (15), it might be important for these cells to generate distinct signals. However, there now appear to be two problems with this argument. First, our simulations of the interactions of activated T cells and mature DCs suggest that human PD-1 will engage ∼4-fold more PD-L1 than PD-L2 molecules due to the low expression of PD-L2 on mature DCs, despite the 3–4-fold lower affinity of PD-L1. Only when the expression of PD-L2 is increased ∼4-fold is the level of accumulation of PD-1·PD-L2 complexes comparable with that of PD-1·PD-L1 complexes. This suggests that signaling at contacts with mature DCs could be dominated by PD-L1. The second issue is that the ∼4-fold higher affinity of human PD-L2 *versus* PD-L1 is not observed in mice, arguing against affinity differences being highly significant (the affinity of the PD-1/PD-L1 interaction is in fact slightly higher in mice: *K_d_* ∼29.8 μm *versus* ∼38.4 μm). One theoretical possibility is that the binding of PD-L1 and PD-L2 induces different conformational rearrangements in PD-1, resulting in distinct types of signals. However, the localized and largely similar effects of the ligands on PD-1 backbone NMR signals, which we found to be restricted to one face only, appear to rule this out (for human PD-1 at least). Overall, the intrinsic signaling properties of PD-L1 and PD-L2 in the mouse seem likely to be identical, suggesting that a capacity to produce differential signals is not the raison d'être of the paired ligands. However, it is also clear that PD-L1 and PD-L2 are not functionally redundant, even in mice. In PD-L1-deficient mice, CD8^+^ T cells spontaneously accumulate in the liver, accelerating hepatocyte damage in an experimental model of autoimmune hepatitis (64), whereas antigen-specific CD8^+^ T cell responses and cytotoxic T lymphocyte activity are diminished in PD-L2-deficient mice (65). At present, these effects cannot be explained by the known biophysical properties of these interactions. It must, however, be acknowledged that we might have failed to mimic the actual patterns of *in vivo* expression of PD-L1/2 in our simulations.

A final matter concerns the likely impact of B7-1/PD-L1 interactions on PD-1, CD28, and CTLA-4 function. The affinity data used to support a competition model (22) were obtained with the use of bivalent forms of the proteins, which it now seems greatly overestimated the strength of the B7-1/PD-L1 interaction. There is little chance of B7-1 and PD-L1 being present at interfaces without other binding competitors, but the affinity of their interaction according to the present study is at least 2-fold and as much as ∼100-fold lower than that of other interactions involving these proteins (*K_d_* of human PD-L1/B7-1 ∼18.8 μm; *K_d_* of human PD-1/PD-L1 ∼7.8 μm; *K_d_* of human CD28·B7-1 ∼4 μm (28); *K_d_* of human CTLA-4·B7-1 ∼0.2 μm (28)). This suggests that B7-1/PD-L1 interactions might have very limited impact. The simulations show, for example, that the expression on T cells of B7-1 (which we were unable to detect) would need to reach a level of >5000 copies/cell (*i.e.* even higher than on mature DCs) in order for the B7-1/PD-L1 interaction to impact PD-1/PD-L1 binding. In the initial study by Butte *et al.* (22), the inhibitory role of the PD-L1/B7-1 interaction was demonstrated by comparing CD28/CTLA-4 double-deficient T cells *versus* CD28/CTLA-4/PD-L1 triple-deficient T cells. Thus the inhibitory role of PD-L1/B7-1 interactions was studied in the absence of “conventional” receptors for B7-1, CTLA-4, and CD28 (*i.e.* in the absence of competition), perhaps exaggerating the physiological importance of B7-1/PD-L1 binding. The effects of this interaction, if any, are likely to be restricted to protein-intrinsic effects on signaling.

## Footnotes

5 J. H. and S. J. D., unpublished data.

## Footnotes

4 The abbreviations used are:

TCR: T cell receptor
DC: dendritic cell
hPD-1: hPD-L1, and hPD-L2, human PD-1, PD-L1, and PD-L2, respectively
mPD-1: mPD-L1, and mPD-L2, mouse PD-1, PD-L1, and PD-L2, respectively
RU: response units
ITC: isothermal titration calorimetry
PDB: Protein Data Bank
SPR: surface plasmon resonance
PE: phycoerythrin.
